# Integrative network analysis identifies potential targets and drugs for ovarian cancer

**DOI:** 10.1186/s12920-020-00773-2

**Published:** 2020-09-21

**Authors:** Tianyu Zhang, Liwei Zhang, Fuhai Li

**Affiliations:** 1 Institute for Informatics (I2), Washington University School of Medicine, Washington University in St. Louis, St. Louis, MO 63130 USA; 2 Dalian University of Technology, Dalian, 116024 China; 3 Department of Pediatrics, Washington University School of Medicine, Washington University in St. Louis, St. Louis, MO 63130 USA

**Keywords:** Ovarian cancer, Core signaling pathways, Network analysis, Drug discovery

## Abstract

**Background:**

Though accounts for 2.5% of all cancers in female, the death rate of ovarian cancer is high, which is the fifth leading cause of cancer death (5% of all cancer death) in female. The 5-year survival rate of ovarian cancer is less than 50%. The oncogenic molecular signaling of ovarian cancer are complicated and remain unclear, and there is a lack of effective targeted therapies for ovarian cancer treatment.

**Methods:**

In this study, we propose to investigate activated signaling pathways of individual ovarian cancer patients and sub-groups; and identify potential targets and drugs that are able to disrupt the activated signaling pathways. Specifically, we first identify the up-regulated genes of individual cancer patients using Markov chain Monte Carlo (MCMC), and then identify the potential activated transcription factors. After dividing ovarian cancer patients into several sub-groups sharing common transcription factors using K-modes method, we uncover the up-stream signaling pathways of activated transcription factors in each sub-group. Finally, we mapped all FDA approved drugs targeting on the upstream signaling.

**Results:**

The 427 ovarian cancer samples were divided into 3 sub-groups (with 100, 172, 155 samples respectively) based on the activated TFs (with 14, 25, 26 activated TFs respectively). Multiple up-stream signaling pathways, e.g., MYC, WNT, PDGFRA (RTK), PI3K, AKT TP53, and MTOR, are uncovered to activate the discovered TFs. In addition, 66 FDA approved drugs were identified targeting on the uncovered core signaling pathways. Forty-four drugs had been reported in ovarian cancer related reports. The signaling diversity and heterogeneity can be potential therapeutic targets for drug combination discovery.

**Conclusions:**

The proposed integrative network analysis could uncover potential core signaling pathways, targets and drugs for ovarian cancer treatment.

## Background

In United States, ovarian cancer is the fifth leading cause of cancer-related death in female [1], which accounts for 2.5% of all cancers in female, whereas, 5% of all cancer death in female [2]. In 2018, there are about 22,000 new cases of ovarian cancer, and 14,000 deaths [2]. The high death rate (< 50% of 5 year survival rate) is mainly because of the late diagnosis and aggressive high grade serous carcinoma [2, 3]. Platinum-based chemotherapy after surgical debulking is the standard treatment for ovarian cancer [4]. However, the cancer recurrence rate is high, and recurred tumors are often platinum resistant [4–6], with complicated mechanism of platinum resistance [7]. Though a few targeted therapies are being evaluated in clinical trials, e.g., VEGF, PARP, EGFR inhibitors [4], some of them are not very successful [4]. Therefore, novel targeted therapies and synergistic drug combinations are needed for ovarian cancer.

On the other hand, comprehensive multi-omics data of ovarian cancer patients have been profiled and analyzed [1, 8]. A set of genetic biomarkers, e.g., TP53, NOTCH, FOXM1, have been identified via association analyses [1]. Also, a few dysfunctional signaling pathways, e.g., MYC, TP53, PI3K/RAS, were be identified in ovarian cancer by mapping multi-omics data, e.g., differentially expressed genes, mutations, copy number variation, and methylation data, to the curated signaling pathways [8]. However, the functional consequence of these biomarkers and cross-talk of complicated signaling pathways in ovarian cancer remain unclear. It is still a challenge to discover effective drugs and synergistic drug combinations [9–12] for ovarian cancer based these valuable knowledge and multi-omics data.

In this study, we aim to systematically investigate potential activated core signaling pathways in ovarian cancer sub-groups by uncovering the up-stream signaling pathways of activated transcription factors (TFs), and identify all available FDA approved drugs targeting on these up-stream signaling and TFs. The combinations of these drugs have the potential to be synergy with standard platinum chemotherapy by disrupting multiple up-stream signaling and their cross-talk. This study will provide a useful reference resource for repositioning effective drugs and drug combinations for ovarian cancer. The rest of the paper is organized as follows. The details of datasets and methods are provided in Section 2. The analysis results are presented in Section 3, followed by a summary in Section 4.

## Methods

### Gene expression data of ovarian cancer and ovarian normal tissue

We download the gene expression (RNAseq - RSEM expected_count (DESeq2 standardized)) data of 427 ovarian cancer samples (from The Cancer Genome Atlas (TCGA) [1]), and 88 ovarian normal samples (from Genotype-Tissue Expression (GTEx) [13]) from the Xena server [14].

### KEGG signaling pathways and regulatory network

To obtain KEGG signaling pathways, the “Pathview” R package [15] was employed to download KGMLs of signaling pathways. Then the “KEGGgraph” R package was used to extract nodes and edges of KEGG signaling pathways from KGMLs [16]. In total, 282 signaling pathways were collected from seven categories: metabolism, genetic information processing, environmental information processing, cellular processes, organismal systems, human diseases, and drug development. The TF-Target regulatory network was downloaded from the supplemental material of reference [17], which was derived from the TF binding site predictions for all target genes from TRANSFAC (v7.4) [18]. In summary, the TF-target regulatory network consists of 230 TFs, 12,733 target genes, and 79,100 TF-Target interactions.

### Drug combination screening data in NCI ALMANAC

This dataset includes screening results of pairwise combinations of 104 FDA-approved anticancer drugs on NCI-60 cancer cell lines (59 cancer cell lines with detailed genomics profiles) [19]. Specifically, ~ 5232 pairwise drug combinations were evaluated in each cancer cell line. Each drug combination was tested at either 9 or 15 dose points for a total of 2,809,671 dose-specific combinations. The detailed definition of synergistic drug combination score was introduced in reference [19].

### Selection of up-regulated genes for each sample

**Fig. 1:**
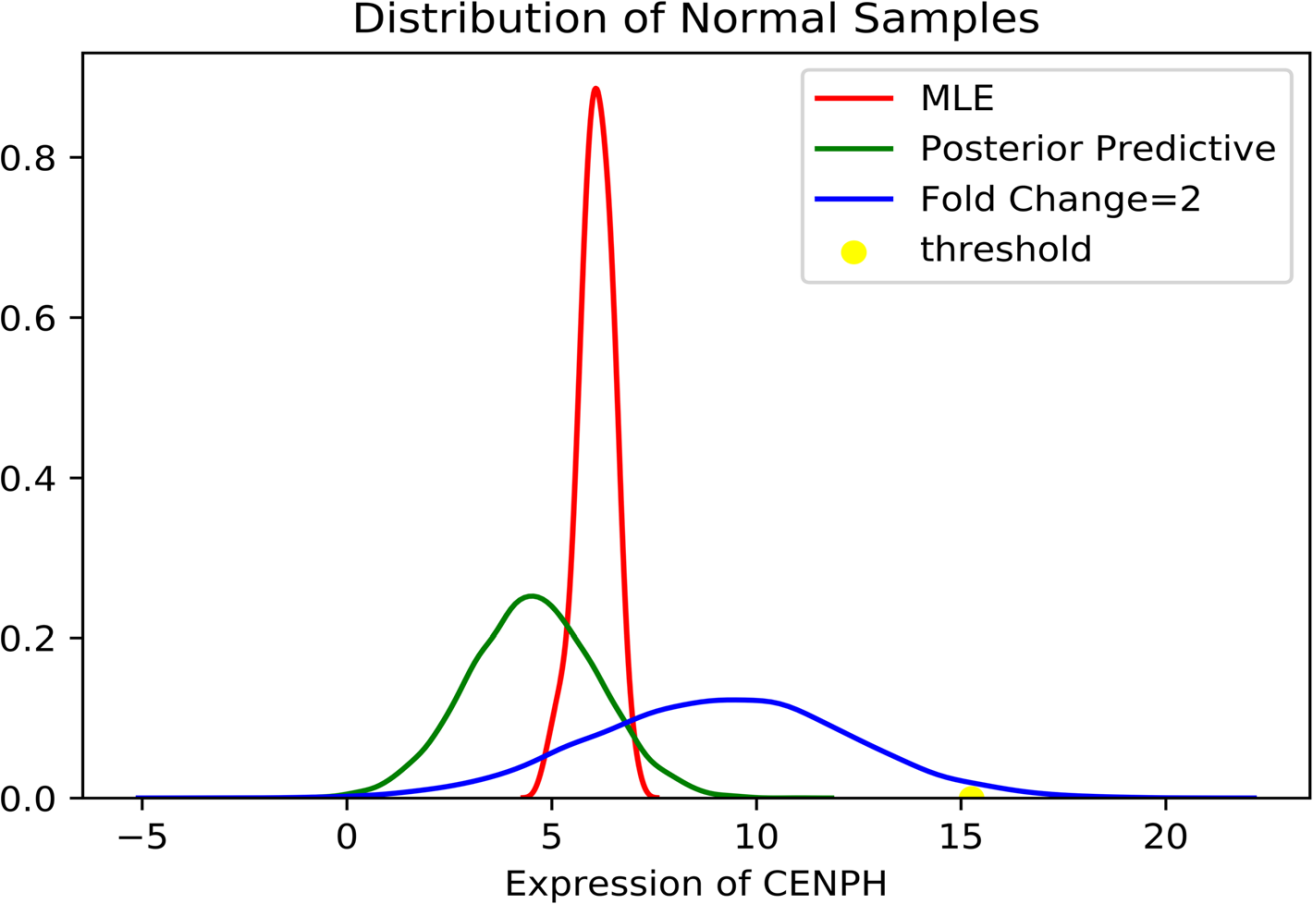
Gene expression distribution of gene “CENPH”

In this study, the GTEx normal ovarian tissue samples were used as normal control versus ovarian cancer tumor samples from TCGA. The simple fold change and *p*-value <= 0.05 (using t test) will result in too many up-regulated genes. The Maximum Likelihood Estimate (MLE) method (see Fig. 1, red probability distribution function (PDF) curve) also generated too many up-regulated genes. Thus, we employ the Markov chain Monte Carlo (MCMC) model to simulate the distribution of gene expression distribution of given genes based on the normal tissues. Let x, D present the gene expression of a given gene and normal tissues respectively.

1 $$ p\left(x|D\right)=\frac{p(xD)}{p(D)}=\frac{\int_{\theta \in \Theta}p\left( xD|\theta \right) d\theta}{p(D)}=\frac{\int_{\theta \in \Theta}p\left(x|\theta \right)p\left(D|\theta \right)}{p(D)} $$

$$ \theta =\left(\mu, {\sigma}^2\right),\kern0.5em \Theta =\left\lfloor -\infty, +\infty \right\rfloor \times \left\lfloor 0,+\infty \right\rfloor, \kern0.5em x:N=\left(\mu, {\sigma}^2\right). $$

We use the conjugate priors for *μ* and*σ*^2^ , which are the Normal distribution and Inverse Gamma distribution: *μ* : *N*(*w*_0_, *v*_0_), *σ*^2^ : *IG*(*a*_0_, *b*_0_).. To get uninformative priors, we set *w*_*0*_ *= 0, v*_*0*_ *= +∞, a*_*0*_ *= 0, b*_*0*_ *= 0.* Since it is hard to calculate eq. (1), we use MCMC method to simulate the distribution. The python package “Pymc3” [20] was employed to conduct the analysis. We set *w*_*0*_ *= 0, v*_*0*_ *= 10*^*4*^*, a*_*0*_ *= 10*^*− 3*^*, b*_*0*_ *= 10*^*− 3*^*.* The MCMC model is better than MLE (see the green PDF curve in Fig. 1), but still too many up-regulated genes will be selected. To further reduce the number of up-regulated genes, we empirically simulate the PDF of random variable y = 2x, and use the PDF of y to calculate the *p*-value of given gene expression in ovarian cancer samples. Specifically, we selected up-regulated genes for each tumor sample with fold change> = 2 and *p*-value<=0.05 (calculated based on the PDF of random variable y). We take the gene “CENPH” as an example to illustrate this analysis. The PDF generated by the MCMC model is more robust than generated by Maximum Likelihood Estimate (MLE) (see Fig. 1). The yellow point is the threshold and area under blue curve on the right of yellow point is about 0.05 (the calculation of *p*-value).

### Identification of activated TFs for individual ovarian cancer patients

The Fisher’s exact test (using hyper-geometric distribution) was used to identify the activated TFs by comparing the number of up-regulated targets vs. the number of all target genes, with the number of all the up-regulated genes vs. the number of all the genes tested. The *p*-value threshold, 0.05, was used to select the activated TFs.

### Sub-grouping analysis using activated TFs

We cluster 427 ovarian cancer samples using the identified activated TFs. We transform *p*-value to 0–1 using 0.05 as a threshold. For categorical data, we use the k-modes method [21] for the sub-grouping analysis.

### Uncovering up-stream signaling of activated TFs

All 282 signaling pathways from KEGG are investigated, and all the signaling cascades from the starting nodes to the activated TFs are extracted using the python package, NetworkX, to extract the up-stream signaling cascades starting from the beginning genes of individual signaling pathways to the given TFs. Then we score each signaling cascades using the average probability of genes (obtained from the MCMC analysis). To control the size of up-stream signaling network, the top 3 signaling cascades are kept.

### Target importance scoring

The impact analysis (IA) evaluates both the topology and dynamics of a signaling pathway by considering the gene expression changes, the direction and type of signaling interaction, and the position and role of every gene in a pathway. A perturbation factor for each gene, PF(*g*_*i*_), is calculated using the impact analysis method [22], as follows:

$$ PF\left({g}_i\right)=\Delta E\left({g}_i\right)+\sum \limits_{j=1}^n{\beta}_{ij}\frac{PF\left({g}_j\right)}{N_{ds}\left({g}_j\right)}, $$

The term ΔE(*g*_*i*_) represents the signed normalized measured gene expression change of gene g_*i*_. The second term is the sum of perturbation factors of direct upstream genes of target gene g_*i*_, normalized by the number of downstream genes of each such gene N_*ds*_(*g*_*j*_). The value of β_*ij*_ quantifies the strength of the interaction between genes g_*j*_ and g_*i*_. We use the probability density of gene expression instead of gene expression, which s will be more accurate considering that the standard deviation of different genes is different.

## Results

### Ovarian cancer samples were clustered into 3 groups based on activated TFs

Using the K-modes method, the 427 ovarian cancer samples were classified into 3 sub-groups (with 100, 172, 155 samples respectively) based on the activated TFs. For each sub-group, there is a center sample, and we use the center sample to characterize each sub-group. In another word, the activated TFs in the center sample were used as the activated TFs for this sub-group.

**Fig. 2:**
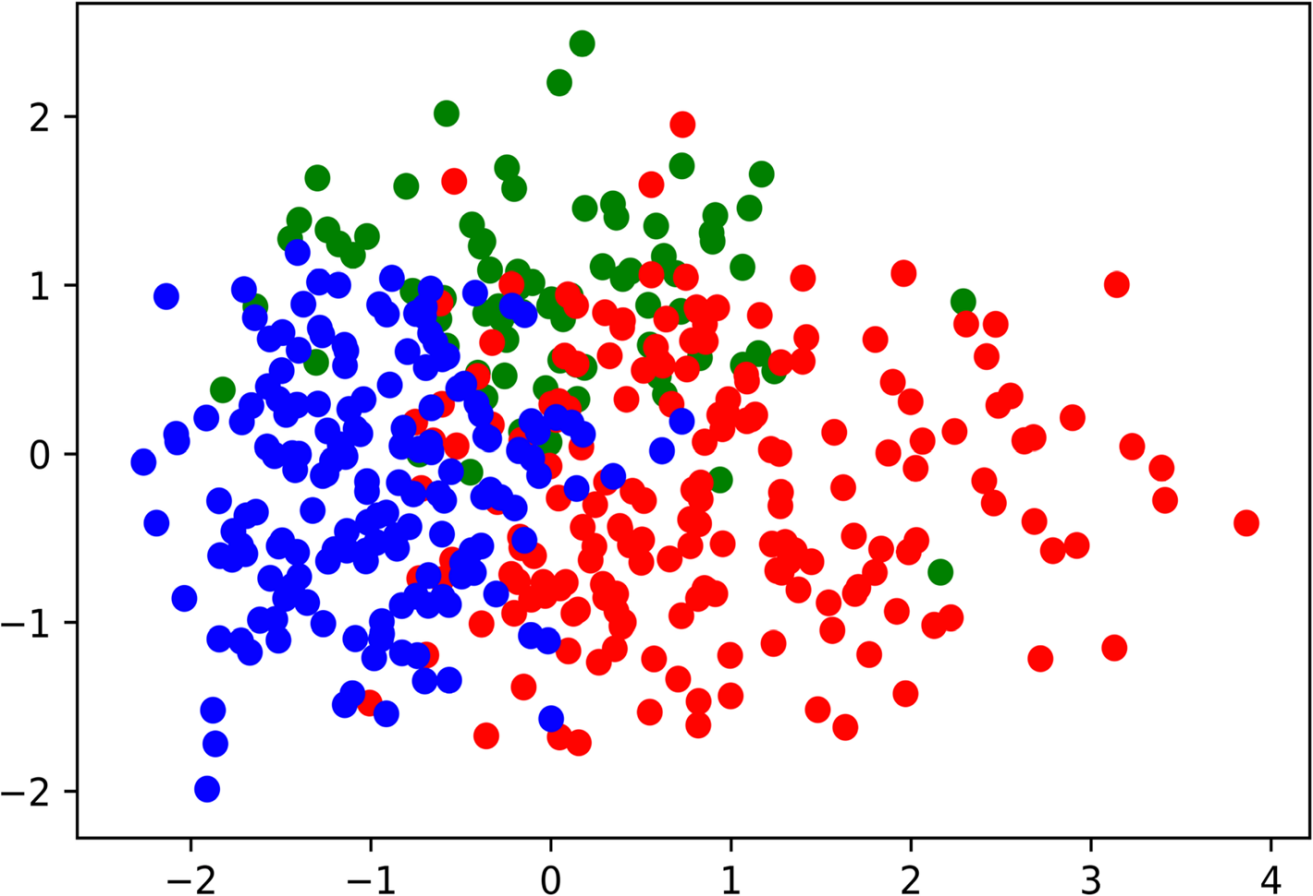
Ovarian cancer samples are clustered into 3 groups based on the activated TFs

For visualization purpose, the principal component analysis (PCA) was employed to reduce the 230 TFs to 2 dimensions (see Fig. 2). In one sub-group (Group 1), 14 TFs were activated: ELK1, FOXF2, NRF1, ETS2, NF.muE1, ADD1, TBP, SP1, GABP, E4F1, TELO2, MYC, YY1, NFE2L2A. Interestingly, these 14 TFs are also activated in the other two groups. **Group 2** and **Group 3** have 26 and 25 TFs respectively. The additional TFs for **Group2** are: AR, ETS1, GABPB1, GFI1, HMG, LHX3, NKX6.2, PAX3, PDX1, PITX2, REST(NRSF), S8. The additional TFs for **Group 3** are: ARNT_MAX, ETS1, FOXN1, FOXO4, GABPB1, LHX3, NFATC2, NFIL3_ATF2, NKX6.2, PAX3, SREBF1.

### Up-stream signaling of activated TFs and related FDA approved drugs

**Fig. 3:**
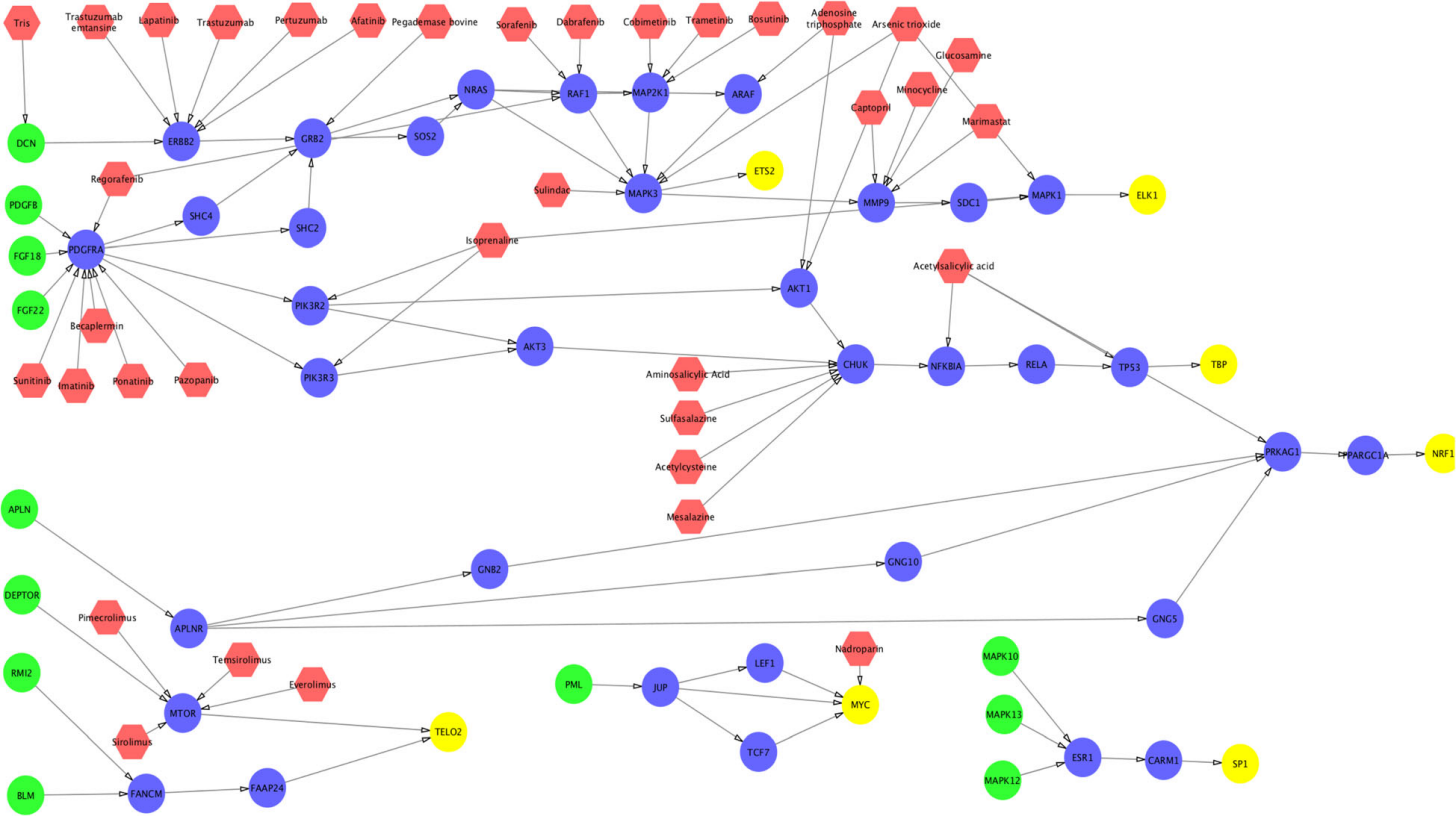
FDA approved drugs targeting on up-stream signaling of activated TFs in Group 1. The color of green, blue, yellow, and red represents signaling starting genes, signaling transduction genes, TFs, and drugs respectively

**Fig. 4:**
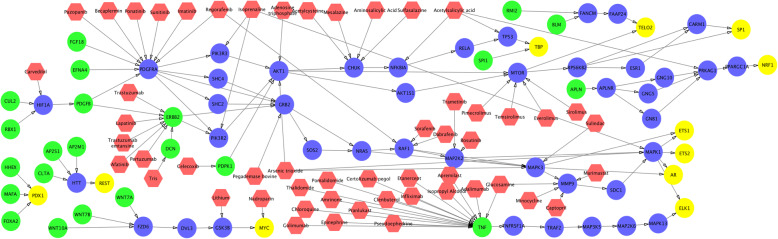
FDA approved drugs targeting on up-stream signaling of activated TFs in group 2. The color of green, blue, yellow, and red represents signaling starting genes, signaling transduction genes, TFs, and drugs respectively

**Fig. 5:**
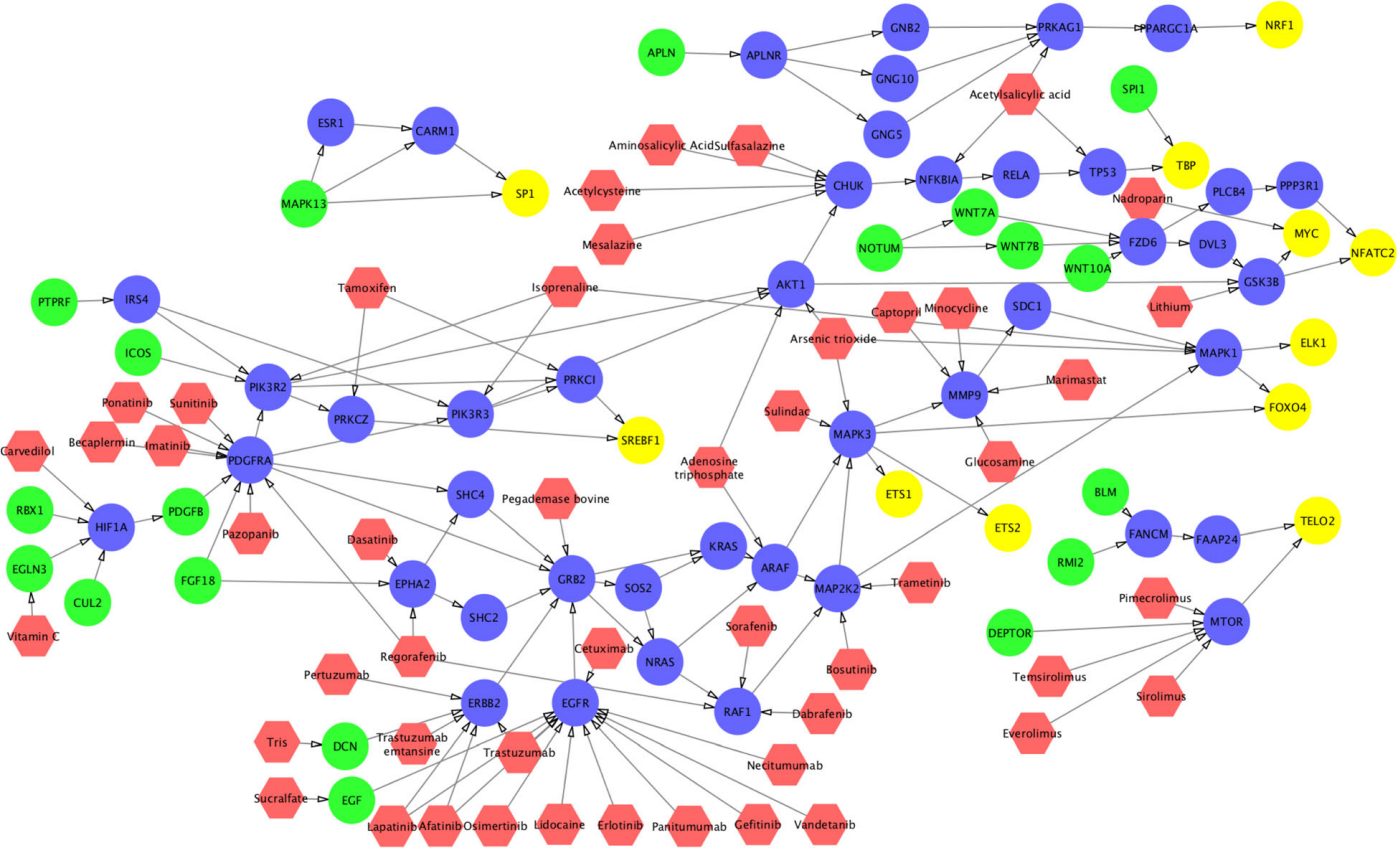
FDA approved drugs targeting on up-stream signaling of activated TFs in group 3. The color of green, blue, yellow, and red represents signaling starting genes, signaling transduction genes, TFs, and drugs respectively

The up-stream signaling of activated TFs are shown in Figs. 3, 4 and 5. As can be seen, multiple important signaling pathways are uncovered, e.g., MYC, WNT, PDGFRA (RTK), PI3K, AKT TP53, and MTOR. This result is consistent with the discoveries in aforementioned references. There are 43 common genes among these 3 sub-groups. We calculated and ranked the perturbation factor of 43 common genes. The top 5 related genes are TBP, MMP9, MYC, MAPK1, MTOR, which might play important roles in ovarian cancer. An interesting finding is MMP9, MYC, MAPK1, MTOR are all in Proteoglycans in cancer. Thus RTK-PI3K-AKT-MTOR can be an important signaling cascade for ovarian cancer. In addition, MTOR actives TP53 by cellular senescence pathway while T53 inhibits MTOR through IGF1/MTOR. Since TP53 is the most frequently altered genes in ovarian cancer, the signaling loop between TP53 and MTOR might be a potential target of novel synergistic drug combinations. Moreover, drug combinations targeting on multiple up-stream signaling and TFs are also potentially synergy to disrupt the activated signaling of ovarian cancer sub-groups.

To investigate potential drugs that can potentially perturb these up-stream signaling networks, we mapped the FDA approved drugs on the signaling networks (see Figs. 3, 4 and 5). The target information was obtained from DrugBank (version 5.0.11) [23]. In total, 66 drugs (red nodes in Figs. 3, 4 and 5) were selected targeting on different targets. Through the literature search, 44 drugs had been reported to treat ovarian cancer (see Table 1). In addition to these single drugs, we investigated effective combinations that appeared in our drug list, and validated in the drug combination screening on NCI 60 ovarian cancer cell lines (the synergy is defined with a threshold score higher than 8) (**see** Table 2). Moreover, we found that the top 10 drug targets of synergistic drug combinations are EGFR, TUBB1, TUBA4A, TUBB, TOP2B, MTOR, TUBB3, CYP19A1, ERS1 and BCL2. TUBB1, TUBA4A, TUBB, TUBB3 and TOP2B are related to cell proliferation. CYP19A1 and ERS1 are related to estrogen. BCL2 is the member of the Bcl-2 family of regulator proteins that regulate cell death. EGFR and MTOR are in PI3K-AKT pathway, and EGFR is one of the upstream of MTOR signaling. The combination of MTOR inhibitors, and EGFR, RTK, PI3K signaling inhibitors might be synergy to inhibit ovarian cancer development.

**Table 1:** FDA approved drugs targeting on upstream signaling of activated transcription factors (TFs)

Drug	Target	Titles of articles related to ovarian cancer treatment
Acetylcysteine	CHUK	N-acetylcysteine potentiates doxorubicin-induced ATM and p53 activation in ovarian cancer cells [24].
Acetylsalicylic acid	TP53, PRKAG1, NFKBIA	Aspirin inhibits growth of ovarian cancer by upregulating caspase-3 and downregulating bcl-2 [25].
Adalimumab	TNF	
Adenosine triphosphate	ARAF, AKT1	
Afatinib	ERBB2, EGFR	Afatinib reverses multidrug resistance in ovarian cancer via dually inhibiting ATP binding cassette subfamily B member 1 [26].
Aminosalicylic acid	CHUK	
Amrinone	TNF	
Apremilast	TNF	
Arsenic trioxide	MAPK1, MAPK3, AKT1	Arsenic Trioxide inhibits the growth of human ovarian carcinoma cell line [27].
Becaplermin	PDGFRA	
Bosutinib	MAP2K1, MAP2K2	Ovarian Cancer Stem Cell Markers: Prognostic and Therapeutic Implications [28].
Captopril	MMP9	Epithelial ovarian cancer: A feasible plan for adjunctive treatment using simultaneous acyclovir, ambrisentan, captopril, disulfiram, fluvoxamine-augmented ramelteon, icatibant, imiquimod peritoneal lavage, and plerixafor [29].
Carvedilol	HIFA1	Cardiovascular complications of antiangiogenic therapy in ovarian cancer patients [30].
Celecoxib	PDPK1	The effect of celecoxib on tumor growth in ovarian cancer cells and a genetically engineered mouse model of serous ovarian cancer [31].
Certolizumab pegol	TNF	
Cetuximab	EGFR	Phase II Trial of Cetuximab and Carboplatin in Relapsed Platinum-Sensitive Ovarian Cancer and Evaluation of Epidermal Growth Factor Receptor Expression: A Gynecologic Oncology Group Study [32].
Chloroquine	TNF	Low concentration of chloroquine enhanced efficacy of cisplatin in the treatment of human ovarian cancer dependent on autophagy [33].
Clenbuterol	TNF	
Cobimetinib	MAP2K1	
Dabrafenib	RAF1	
Dasatinib	EPHA2	Activity of the multikinase inhibitor dasatinib against ovarian cancer cells [34].
Epinephrine	TNF	
Erlotinib	EGFR	Erlotinib or gefitinib for the treatment of relapsed platinum pretreated non-small cell lung cancer and ovarian cancer: a systematic review [35].
Etanercept	TNF	Study of etanercept, a tumor necrosis factor-alpha inhibitor, in recurrent ovarian cancer [36].
Everolimus	MTOR	Effective use of everolimus as salvage chemotherapy for ovarian clear cell carcinoma: a case report [37].
Gefitinib	EGFR	Gefitinib (ZD1839) increases the efficacy of cisplatin in ovarian cancer cells [38].
Glucosamine	TNF, MMP9	
Golimumab	TNF	
Imatinib	PDGFRA	Imatinib mesylate (Gleevec) inhibits ovarian cancer cell growth through a mechanism dependent on platelet-derived growth factor receptor alpha and Akt inactivation [39].
Infliximab	TNF	Infliximab, a humanised anti-TNF-a monoclonal antibody, exhibits biological activity in the ovarian tumor microenvironment in patients [40].
Isoprenaline	PIK3R3, PIK3R2, MAPK1	Targeted anti-vascular therapies for ovarian cancer: current evidence [41].
Isopropyl alcohol	TNF	
Lapatinib	ERBB2, EGFR	A phase II evaluation of lapatinib in the treatment of persistent or recurrent epithelial ovarian or primary peritoneal carcinoma: a gynecologic oncology group study [32].
Lidocaine	EGFR	Lidocaine inhibits the invasion and migration of TRPV6-expressing cancer cells by TRPV6 downregulation [42].
Lithium	GSK3B	Lithium and inhibition of GSK3β as a potential therapy for serous ovarian cancer [43].
Marimastat	MMP9	Marimastat (BB2516): Current status of development [44].
Mesalazine	CHUK	
Minocycline	MMP9	Minocycline inhibits malignant ascites of ovarian cancer through targeting multiple signaling pathways [45].
Nadroparin	MYC	
Necitumumab	EGFR	
Osimertinib	EGFR	
Panitumumab	EGFR	Targeting the Epidermal Growth Factor Receptor in Epithelial Ovarian Cancer: Current Knowledge and Future Challenges [46].
Pazopanib	PDGFRA	Incorporation of Pazopanib in Maintenance Therapy of Ovarian Cancer [47].
Pegademase bovine	GRB2	
Pertuzumab	ERBB2	A randomized phase II study evaluating the combination of carboplatin-based chemotherapy with pertuzumab versus carboplatin-based therapy alone in patients with relapsed, platinum-sensitive ovarian cancer [48].
Pimecrolimus	MTOR	Topical pimecrolimus inhibits high-dose UVB irradiation-induced epidermal Langerhans cell migration, via regulation of TNF-a and E-cadherin [49].
Pomalidomide	TNF	
Ponatinib	PDGFRA	Ponatinib Shows Potent Antitumor Activity in Small Cell Carcinoma of the Ovary Hypercalcemic Type (SCCOHT) through Multikinase Inhibition [50].
Pranlukast	TNF	
Pseudoephedrine	TNF	
Regorafenib	EPHA2, RAF1, PDGFRA	301P Interim Analysis of A Single-Arm Phase 2 Clinical Trial of Regorafenib in Patients with Epithelial Ovarian Cancer [51].
Sirolimus	MTOR	Rapamycin by itself and additively in combination with carboplatin inhibits the growth of ovarian cancer cells [52].
Sorafenib	RAF1	Activity of sorafenib in recurrent ovarian cancer and primary peritoneal carcinomatosis: a gynecologic oncology group trial [53].
Sucralfate	EGF	
Sulfasalazine	CHUK	Sulfasalazine Inhibits IL-2 Expression in Ovarian Cancer Cells [54].
Sulindac	MAKP3	The conventional nonsteroidal anti-inflammatory drug sulindac sulfide arrests ovarian cancer cell growth via the expression of NAG-1/MIC-1/GDF-15 [55].
Sunitinib	PDGFRA	Autophagy Inhibition Enhances Sunitinib Efficacy in Clear Cell Ovarian Carcinoma [56].
Tamoxifen	PRKCZ, ESR1, PRKCI	The efficacy of tamoxifen in patients with advanced epithelial ovarian cancer [57].
Temsirolimus	MTOR	Temsirolimus in women with platinum-refractory/resistant ovarian cancer or advanced/recurrent endometrial carcinoma. A phase II study of the AGO-study group (AGO-GYN8) [58].
Thalidomide	TNF	Thalidomide and lenalidomide for recurrent ovarian cancer: A systematic review of the literature [59].
Trametinib	MAP2K1, MAP2K2	The mTORC1/2 Inhibitor AZD8055 Strengthens the Efficiency of the MEK Inhibitor Trametinib to Reduce the Mcl-1/[Bim and Puma] ratio and to Sensitize Ovarian Carcinoma Cells to ABT-737 [60].
Trastuzumab	ERBB2, EGFR	Trastuzumab Sensitizes Ovarian Cancer Cells to EGFR-targeted Therapeutics [61].
Trastuzumab emtansine	ERBB2	Superior in vitro and in vivo activity of trastuzumab-emtansine (T-DM1) in comparison to trastuzumab, pertuzumab and their combination in epithelial ovarian carcinoma with high HER2/neu expression [62].
Tris	DCN	Synergism from Combinations of tris(benzimidazole) monochloroplatinum(II) Chloride with Capsaicin, Quercetin, Curcumin and Cisplatin in Human Ovarian Cancer Cell Lines [63].
Vandetanib	EGFR	Vandetanib, designed to inhibit VEGFR2 and EGFR signaling, had no clinical activity as monotherapy for recurrent ovarian cancer and no detectable modulation of VEGFR2 [64].
Vitamin c	EGLN3	The Effect of Intravenous Vitamin C on Cancer- and Chemotherapy-Related Fatigue and Quality of Life [65].

**Table 2:** Validated synergistic drug combinations in NCI-60

Drug 1	Drug 2	Target 1	Target 2	Score	Cell line
Erlotinib	Dasatinib	EGFR	EPHA2	34.44	IGROV1
Gefitinib	Dasatinib	EGFR	EPHA2	23.22	IGROV1
Vandetanib	Dasatinib	EGFR	EPHA2	18.44	IGROV1
Dasatinib	Tamoxifen	EPHA2	PRKCZ, PRKCI	16.78	IGROV1
Lapatinib	Sirolimus	ERBB2, EGFR	MTOR	14.00	IGROV1
Vandetanib	Everolimus	EGFR	MTOR	13.56	IGROV1
Celecoxib	Dasatinib	PDPK1	EPHA2	13.11	IGROV1
Lapatinib	Everolimus	ERBB2, EGFR	MTOR	12.44	IGROV1
Sirolimus	Tamoxifen	MTOR	PRKCZ, PRKCI	11.67	IGROV1
Lapatinib	Dasatinib	ERBB2, EGFR	EPHA2	11.00	IGROV1
Gefitinib	Everolimus	EGFR	MTOR	10.78	IGROV1
Dasatinib	Imatinib	EPHA2	PDGFRA	10.56	IGROV1
Celecoxib	Vandetanib	PDPK1	EGFR	9.11	IGROV1
Sirolimus	Vandetanib	MTOR	EGFR	8.78	IGROV1
Erlotinib	Tamoxifen	EGFR	PRKCZ, PRKCI	8.33	IGROV1
Dasatinib	Tamoxifen	EPHA2	PRKCZ, PRKCI	11.33	OVCAR-3
Sirolimus	Everolimus	MTOR	MTOR	9.56	OVCAR-3
Celecoxib	Dasatinib	PDPK1	EPHA2	9.44	OVCAR-3
Thalidomide	Dasatinib	TNF	EPHA2	8.89	OVCAR-3
Sirolimus	Gefitinib	MTOR	EGFR	10.56	OVCAR-4
Gefitinib	Everolimus	EGFR	MTOR	9.44	OVCAR-4
Lapatinib	Sirolimus	ERBB2, EGFR	MTOR	9.44	OVCAR-4
Vandetanib	Tamoxifen	EGFR	PRKCZ, PRKCI	9.33	OVCAR-5
Dasatinib	Tamoxifen	EPHA2	PRKCZ, PRKCI	14.78	SK-OV-3
Everolimus	Tamoxifen	MTOR	PRKCZ, PRKCI	11.78	SK-OV-3
Lapatinib	Dasatinib	ERBB2, EGFR	EPHA2	10.11	SK-OV-3
Celecoxib	Dasatinib	PDPK1	EPHA2	9.56	SK-OV-3
Gefitinib	Dasatinib	EGFR	EPHA2	9.11	SK-OV-3

Moreover, we investigated the difference of activated core signaling pathways among these 3 sub-groups. The unique TFs appeared in the core signaling pathways in Group 2 and Group 3 are: PDX1, REST and AR; and ROXO4, SREB F1, NFATC2 respectively. In upstream signaling genes, PML, LEF1, MAPK12, FGF22, JUP, AKT3, MAPK10, MAP2K1, TCF7 are the unique genes for Group1. The CLTA, AR, RPS6KB2, AP2S1, AKT1S1, FOXA2, PDPK1, HTT, MAP2K6, TNFRSF1A, TNF, GNB1, MAFA, TRAF2, REST, HHEX, EFNA4, MAP3K5, PDX1 and AP2M1 are unique genes for Group2. The FOXO4, PLCB4, NFATC2, IRS4, KRAS, PRKCI, PTPRF, ICOS, EGLN3, NOTUM, PPP3R1, SREBF1, EPHA2, EGFR, EGF and PRKCZ are the unique genes for Group3. For Group 1, MAPK10, AKT3, FGF22 are in MAPK signaling pathway and RAS signaling pathway, and PML, JUP, LEF1, TCF7 are signaling cascades linking to MYC. For Group2, the signaling cascade from TNF to p38 is an upstream of p53. For Group3, many genes appeared in RAP1 signaling pathway. For the drugs listed in Table 1, for example, Celecoxib, Chloroquine, Etanercept, Infliximab and Thalidomide targets on unique Group2 genes; and Cetuximab, Dasatinib, Erlotinib, Gefitinib, Lidocaine, Necitumumab, Osimertinib, Panitumumab, Sucralfate, Tamoxifen, Vandetanib, Vitamin C targets on Group 3 unique genes. The signaling diversity and heterogeneity can be potential therapeutic targets for drug combination discovery.

## Discussion

Ovarian cancer is the fifth leading cause of cancer-related death among women, and the 5-year survival rate is fewer than one half. Though a set of biomarkers and signaling pathways have been identified to be associated with ovarian cancer, the functional consequence of these biomarkers and signaling pathways remain unclear. Moreover, there is a lack of effective targeted therapies for ovarian cancer, especially for the platinum resistant ovarian cancer. In this study, we analyzed the gene expression data of ovarian cancer samples and ovarian normal tissues via network analysis. We aim to systematically explore the activated signaling pathways of individual ovarian cancer patients and sub-groups, and identify potential targets and drugs that are able to disrupt the core signaling pathways. There are still several limitations of the study. First, in addition to gene expression, mutation, methylation, and copy number variation data should be integrated in the network analysis to uncover the TFs, and up-stream signaling. Second, the signaling cross-talk among these up-streams are not investigated, which might be responsible for drug resistance. In the future, we will also investigate the signaling network and TFs of platinum resistant ovarian cancer samples; and conduct the network-based drug repositioning approaches [66, 67] to reposition drugs [68, 69] and drug combinations [70] for ovarian cancer treatment.

## Conclusions

The purpose of this study is to systematically uncover potential activated core signaling pathways in ovarian cancer using integrative network analysis. We identified about 37 activated TFs from three sub-groups of ovarian cancer, as well as a set of up-stream signaling pathways linking to these TFs, e.g., WNT, TP53, MYC, AKT, RAS, mTOR, PDGFRA signaling pathways. In addition, 66 FDA approved drugs were identified targeting on the uncovered core signaling pathways. Forty-four drugs had been reported in ovarian cancer related reports. Combinations of these drugs could be potentially synergy to disrupt the cross-talk of multiple activated signaling pathways and TFs for better ovarian therapy. These uncovered signaling networks, TFs and drugs can be used as reference resources to support biomedical studies in ovarian cancer.

## Data Availability

Gene expression data is available at: https://xenabrowser.net/datapages/?dataset=TCGA-GTEx-TARGET-gene-exp-counts.deseq2-normalized.log2&host=https%3A%2F%2Ftoil.xenahubs.net). The TF-Target interactions, derived from TRANSFAC database, are third party data and are available at: https://genome.cshlp.org/content/24/11/1869/suppl/DC1 NCI-60 Drug combination screening data is available at: https://dtp.cancer.gov/discovery_development/nci-60/ Drug-Target interactions are derived from DrugBank: https://www.drugbank.ca/releases/latest

## Abbreviations

TCGA: The cancer genome atlas
GTEx: Genotype-tissue expression
KEGG: Kyoto encyclopedia of genes and genomes
TF: Transcription factor
MCMC: Markov chain Monte Carlo
ALMANAC: A large matrix of anti-neoplastic agent combinations
